# Simple Perylene Diimide Cyclohexane Derivative With Combined CPL and TPA Properties

**DOI:** 10.3389/fchem.2020.00306

**Published:** 2020-04-21

**Authors:** Pablo Reine, Ana M. Ortuño, Inês F. A. Mariz, Maria Ribagorda, Juan M. Cuerva, Araceli G. Campaña, Emerlinda Maçôas, Delia Miguel

**Affiliations:** ^1^Departamento de Química Orgánica, Facultad de Ciencias, Unidad de Excelencia de Química Aplicada a Biomedicina y Medioambiente (UEQ), Universidad de Granada, Granada, Spain; ^2^Centro de Química Estrutural, Instituto Superior Técnico, Universidade de Lisboa, Lisbon, Portugal; ^3^Departamento de Química Orgánica, Facultad de Ciencias, C.U. Cantoblanco, Universidad Autónoma de Madrid, Madrid, Spain; ^4^Departamento de Fisicoquímica, Facultad de Farmacia, UEQ, Universidad de Granada, Granada, Spain

**Keywords:** perylene diimide (PDI), electronic circular dichroism (ECD), circularly polarized luminiscence (CPL), two-photon absorption, non-linear emission

## Abstract

In this work we describe the linear and non-linear (chiro)optical properties of an enantiopure bis-perylenediimide (PDI) cyclohexane derivative. This compound exhibits upconversion based on a two-photon absorption (TPA) process with a cross-section value of 70 GM together with emission of circularly polarized luminescence (CPL), showing a g_lum_ in the range of 10^−3^. This simple structure represents one of the scarce examples of purely organic compounds combining both TPA and CPL responses, together with large values of molar absorptivity and fluorescence quantum yield with emission in the 500–600 nm. Self-assembly induced by introduction of a poor solvent allows for a spectacular shift of the emission into the near-infrared (NIR, 650–750 nm) by formation of well-defined rotationally displaced dimers. Therefore, we are here presenting a versatile platform whose optical properties can be simply tuned by self-assembly or by functionalization of the electron-deficient aromatic core of PDIs.

## Introduction

Organic materials presenting circularly polarized luminescence (CPL) (Riehl and Richardson, 1977; Kumar et al., 2015; Sanchez-Carnerero et al., 2015; Longhi et al., 2016; Tanaka et al., 2018) have recently emerged as promising candidates for advanced optical applications (Zinna et al., 2015; Han et al., 2018; Shi et al., 2018; Zheng et al., 2018; Burrezo et al., 2019; David et al., 2019; Jiménez et al., 2019; Pop et al., 2019; Yang and Zhong, 2019). Thus for example, CPL emitters have been proposed as chiroptical sensors (Staszak et al., 2019), for smart sensing methodologies (Imai et al., 2018; Reine et al., 2018a,b; Zinna et al., 2019), to encode information in light (Andréassons and Pischel, 2018), in patterning processes using security inks (Andres et al., 2014), or constituents of CPL organic light-emitting diodes OLEDs (Brandt et al., 2016; Di Nuzzo et al., 2017). This property is characterized by the preferential emission of left (I_*L*_) or right handed (I_*R*_) circularly polarized light with respect to the entire emission (I_*L*_+I_*R*_)/2, being usually described by the dimensionless Kuhn factor of the emission, *g*_*lum*_ = 2(I_*L*_-I_*R*_)/(I_*L*_+I_*R*_). For simple organic molecules (SOMs), these values usually range from 0.01 to 0.0001, being scarce values higher than 0.01 (Sato et al., 2017; Ito et al., 2018; Takaishi et al., 2018, 2019, 2020; Han et al., 2019; Reine et al., 2019; Schaack et al., 2019; Zheng et al., 2019).

Although the development of this technique is approaching its maturity, the coupling of CPL with other types of optical response can open new avenues in the design, fabrication and application of optical materials. In particular, the coupling of CPL with non-linear excitation opens the possibility of obtaining higher energy emission from low energy photons. Two-photon absorption (TPA) is a non-linear optical property that depends on the third-order optical susceptibility (χ^3^) and it scales with the square of the light intensity used in the excitation process (He et al., 2008). In a two-photon process, low energy photons can cooperatively excite the chromophore, yielding an excited state indistinguishable of that obtained by a one photon excitation. The use of low energy photons (usually NIR light) and the non-linear dependence of the excitation offers the possibility of light actuation at larger penetration depths (e.g., deeper penetration in biological tissue) and intrinsic localization in space. Thus, the TPA process is routinely applied in photolithography to obtain 3D nanopatterning with high aspect ratio. It also finds widespread application in optical imaging of 3D-biological samples in biomedical research, with particular relevance in the study of neuronal activity in awake, behaving animals over long periods of time (Lecoq et al., 2019). Many different types of organic chromophores have been explored in an attempt to improve the TPA properties, from SOM, to polymers, nanoparticles and composites tailored for diverse applications (Zou et al., 2011; Marcelo et al., 2015; Mariz et al., 2015; Santos et al., 2018). The non-linear excitation of a chiral chromophore opens the additional possibility of observing upconverted CPL. Therefore, some efforts have been carried out to combine TPA and CPL (TPCPL) in the same molecule. To our knowledge, very few examples have been described including chiral nanographenic SOMs developed by our group (Cruz et al., 2018a,b, 2019) and chiral Cd(II) 1D structures (Deng et al., 2019). It is remarkable that, up to the moment, there is just one photonic system enabling TPA-induced CPL based on chiral perovskites (Chen et al., 2019).

The combination of TPA and CPL is a promising field of research that will unfold through carefully planned systematic studies. Many opportunities exist for the development of new and simple architectures combining reasonable *g*_*lum*_ values and TPA cross sections (σ_2_) together with good quantum yields (Φ_F_). Among such possibilities simple organic molecules (SOMs) are appreciated owing to their high solubility and processability (Kumar et al., 2015; Sanchez-Carnerero et al., 2015; Longhi et al., 2016; Tanaka et al., 2018).

For the sake of simplicity, we propose as a new approach a chiral arrangement of achiral PDIs to take advantages of the intrinsic photophysical properties of such chromophores. Following a similar strategy, pyrenes have been extensively used in the context of CPL (Hashimoto et al., 2016; Takaishi et al., 2018; Ohishi and Inouye, 2019). Nevertheless, the possibility of forming excimer inter- or intramolecularly drops the quantum yield of the emission. Moreover, simple pyrenes cannot be excited using the most common excitation sources providing high peak power femtosecond pulses in the NIR (700–1,000 nm) that are necessary for the observation of TPA. On the other hand, PDIs are remarkable emitters, with near unit emission yield in all common solvents including aliphatic, aromatic, chlorinated, and dipolar solvents. Their emission wavelength can be tuned via functionalization of either the electron-deficient aromatic core or the imide units. The imide substituents can be used to control their self-assembly into a plethora of supramolecular architectures (Wurthner et al., 2016). It is noteworthy that PDIs (Kawai et al., 2007; Ikeda et al., 2012) and also the smaller analogs napthtalene diimides (Salerno et al., 2017; Keshri et al., 2020) have been successfully used in the context of CPL-SOMs with high quantum yields in solution and/or aggregated forms (Tsumatori et al., 2010).

Different types of chiral scaffolds have been used to place the achiral chromophores in a chiral environment. Remarkable examples are binaphtyl-type skeletons (Hara et al., 2019; Takaishi et al., 2019), dibenzo[b,d]furane (Ito et al., 2017), cyclophanes (Morisaki et al., 2014; Liang et al., 2019; Wang et al., 2020), or [n]-helicenes (Dhbaibi et al., 2020). In this work, we considered as chiral scaffold enantiopure and commercially available (*1R,2R*)-(-)-1,2-diaminecyclohexane for its simplicity and its easy functionalization. This chiral unit has been recently used in the design of CPL emitters based on naphthalimides (Wang et al., 2019) and efficient CPL-OLEDs based on thermally activated delayed fluorescent enantiomers of aromatic-imides (Li et al., 2018).

**Figure 1:**
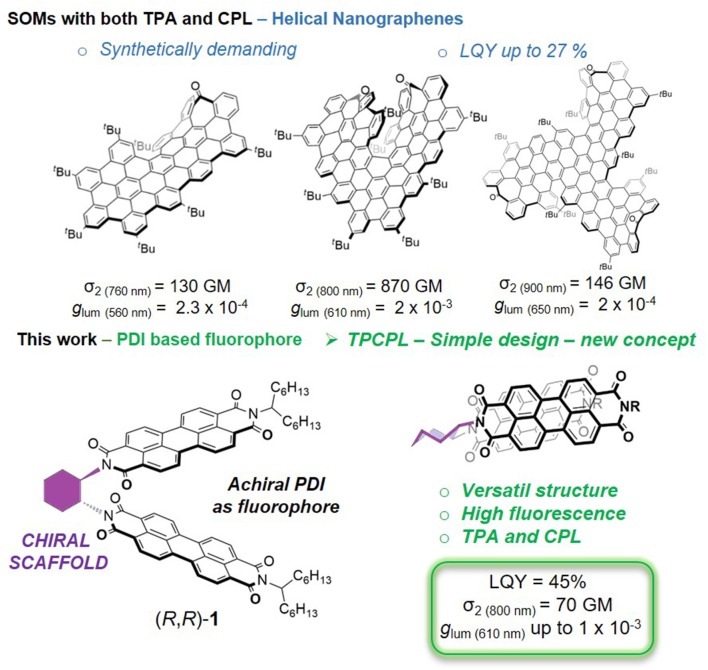
SOMs exhibiting both TPA and CPL responses. **(Top)** Reported examples. **(Bottom)** This work.

Herein we present the interesting linear and non-linear (chiro)optical properties of compound (*R,R*)-**1** (Figure 1) built upon the combination of the chiral diamine and a PDI derivative. This compound can be easily prepared following known procedures (Che et al., 2007; Park et al., 2017). We show that, despite its simple structure, it can compete in terms of quantum yields, *g*_*lum*_ and even TPA cross sections with previously described TPCPL emitters requiring a more demanding synthetic approach.

## Results and Discussion

**Figure 2:**
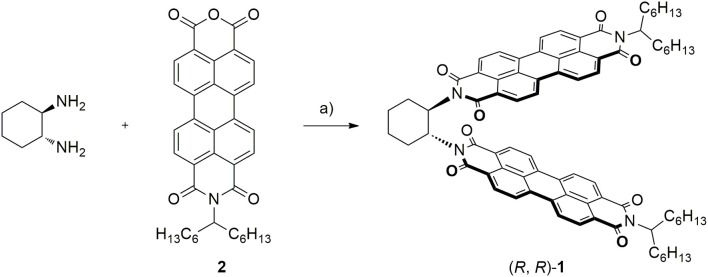
Synthetic route yielding (*R,R*)-**1**. Reagents and conditions: a) (*1R, 2R*)-(–)-1,2-Cyclohexanediamine (1 Eq.), **2** (2.2 Eq), Imidazole as solid solvent (350 Eq.), 140°C, 1 h (27%).

Compound (*R,R*)-**1** was prepared by direct condensation between anhydride **2** and commercially available (*1R,2R*)-(–)-1,2-diaminecyclohexane with good yields (Figure 2). Precursor **2** can be synthetized using a known protocol (Che et al., 2007) from perylene diimide and the corresponding solubilizing chains. At this point we checked some solubilizing chains, finding that at least 13 carbon atoms are required to ensure a reasonable solubility in all the solvents studied.

**Figure 3:**
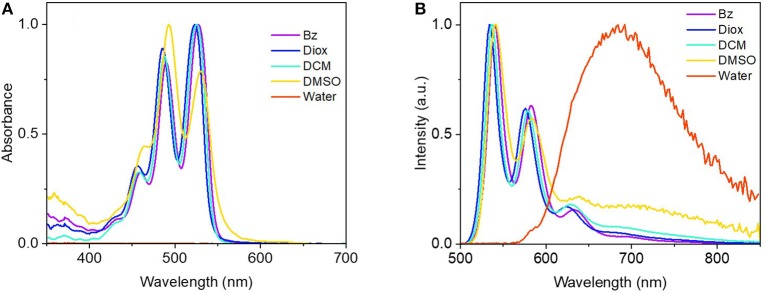
Normalized UV-vis absorbance **(A)** and fluorescence spectra [**(B)**, λ_exc_ = 485 nm] of (*R,R*)-**1** in an illustrative set of solvents of increasing polarity (Bz, benzene; Diox, dioxane; DCM, dichloromethane; DMSO, dymethylsulfoxide) at a concentration of ca. 10^−6^M. Additional solvents are shown in Figure S4. Note that the absorption of a (*R,R*)-**1** suspension in water is not clearly observed due to precipitation and formation of dispersed aggregates.

With compound (*R,R*)-**1** fully characterized by means of ^1^H-NMR, ^13^C-NMR and HRMS (Figures S1, S2), we then analyzed the corresponding linear optical properties in different solvents covering a wide range of polarity, in order to evaluate a possible solvatochromic effect. As it can be seen in Figure 3A (for other solvents see Figure S4), with the exception of water, a similar shape of the absorbance spectra was observed in all solvents. Three main vibronic bands are observed centered at ≈ around 455, 485, and 525 nm characteristic of the PDI units, thus confirming that the inclusion of short aliphatic chains does not alter the spectroscopical properties of PDI (Langhals, 2005; Kawai et al., 2007). The absorbance maximum appeared around 525 nm with no obvious polarity dependent solvatochromic effect from benzene (Bz) to dimethylsulfoxide (DMSO). The molar absorptivity remained almost constant at 1 × 10^5^ M^−1^cm^−1^ as the polarity of the solvent increases from benzene to dimethylformamide (DMF) (Figure S3). In more polar solvents (DMSO and acetonitrile) the molar absorptivity drops considerably and the relative intensity of the vibronic bands is significantly altered suggesting the existence of aggregation. Except from water, all organic solvents presented two main emission peaks with well-defined maxima at ≈ 530 and 580 nm. Similarly to the absorption spectra, the fluorescence spectra show no obvious polarity dependent solvatochromic effect (emission maxima between 530 nm for acetonitrile and acetone and 540 nm for benzene, toluene and DMSO). Although in most of the solvents the emission is again typical of monomeric PDI, we observed an additional broad and unstructured band in the NIR in more polar solvents as DMSO, but also in tetrahydrofuran (THF) and acetone in Figure S4. This band is attributed to the formation of aggregates or excimer-like interactions (Figure 3B). When (*R,R*)-**1** is directly suspended in water most of the compound precipitates and a broad unstructured emission band centered at 690 nm is observed due to dispersed aggregates in solution. No clear absorption is directly observed but the corresponding excitation spectrum shows again a broad and unstructured feature centered at 500 nm (Figure S5). For the sake of completeness, the influence of the excitation wavelength in the emission spectra is also shown for dioxane (Diox) and DMSO in Figure S5. For dioxane the excitation spectrum has a clear vibronic structure, it overlaps perfectly with the absorption spectrum and it is independent on the emission wavelength showing that all the compound is in its monomeric form. For DMSO, the excitation spectrum has also a clear vibronic structure but it depends on the emission wavelength and it does not have a perfect overlap with the absorption spectrum. The excitation maximum appears at 528 nm when collected at typical wavelengths of monomeric emission (574 nm) and it appears blue-shifted to 492 nm for emission collected in the aggregate band at 690 nm. Dioxane and water feature extreme cases where all the compound is either monomeric or aggregated in higher-order aggregates. DMSO is an intermediate situation where we have contributions from both monomeric and different types of aggregates.

Fluorescence quantum yields measured for all solvents show a general trend with higher values in less polar solvents (40-60% in Bz, Tol, Diox, CHCl_3_, DCM) and considerably lower values in more polar solvents (5–20% in acetone, DMF, ACN and DMSO) as shown in Figure S3.

**Figure 4:**
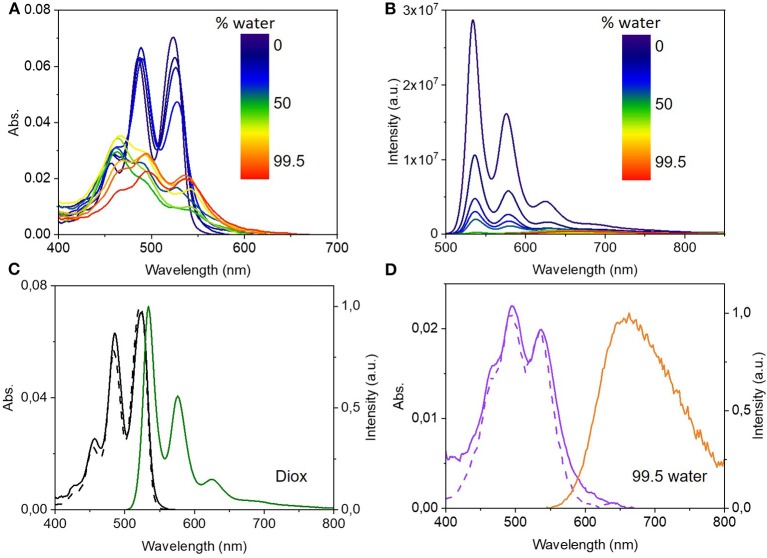
Top: UV-Vis absorbance **(A)** and fluorescence spectra (λ_exc_ = 535 nm) **(B)** of (*R,R*)-**1** in dioxane:water mixtures with increasing water percentage from 0 to 99.5%. Bottom: UV-Vis absorption (black and violet lines), excitation (dashed lines) and fluorescence emission spectra (green and orange lines, λ_exc_ = 535 nm) of (*R,R*)-**1** in 0 vol% **(C)** and 99.5 vol% **(D)** water content in dioxane.

In an attempt to control the self-assembly of (*R,R*)-**1**, diluted solutions of compound (*R,R*)-**1** (*ca* 10^−6^ M) were prepared in different dioxane: water mixtures ranging from 0 to 99.5% water (pure water was avoided due to the partial insolubility of compound (*R,R*)-**1**). Since the compound is monomeric in dioxane and the solvent is completely miscible in water, the systematic addition of the poor solvent, while keeping the concentration in the micromolar range, can lead to the formation of lower order aggregates. As can be seen in Figures 4A,B there is a clear difference in both absorption and emission spectra not only in the wavelength maxima but mainly in the ratio and the intensity of the bands. The increased amount of water promotes a decrease of the intensity of both absorption and emission bands. The integrated absorption drops by almost 70% (Figure 4A) while the integrated emission drops by more than one order of magnitude (Figure 4B). Figures 4C,D illustrate the significantly different behavior of the initial pure dioxane solution featuring the monomer and the 99.5% water solution featuring the aggregate. Interestingly, at high water percentages (>94%) we observe the formation of a well-defined aggregate with a clear vibronic structure in the absorption (Figure 4D) and a broad unstructured emission centered at 690 nm. The excitation spectrum does not depend on the emission wavelength and the emission is also excitation-wavelength independent. These observations confirm that there is only one lower order aggregate in solution, which we assign to a rotationally displaced dimer based on the similarities between its absorption spectrum and that of a model PDI derivative with anti-cooperative supramolecular self-assembly into dimers (Gershberg et al., 2016). Additionally, we observed an increase in the absorption and emission intensity of the dimer with the concentration of (*R,R*)-**1** in solutions with more than 94% of water (Figure S6), which excludes both the possibility of intramolecular aggregation in the ground state and the formation of excimers.

**Figure 5:**
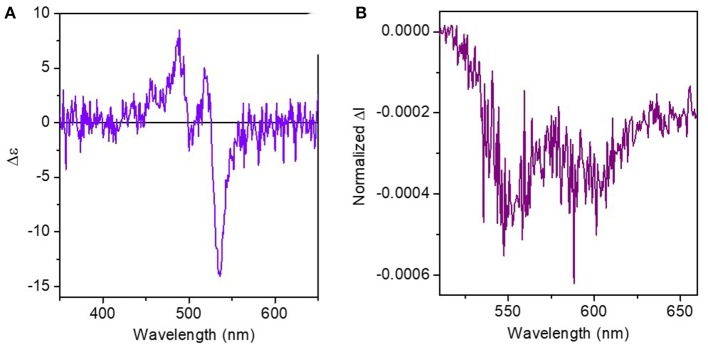
**(A)** ECD and **(B)** CPL spectra of compound (*R,R*)-**1** in dioxane at *ca*. 10^−5^ M.

Chiroptical properties (ECD and CPL) were also recorded in 0 vol% and 99.5 vol% water content dioxane solutions. In neat dioxane, ECD spectra exhibits three main bands at 490, 520, and 540 nm attributed to the PDI skeleton and a value of g_abs_ = 1 × 10^−3^(Figure 5A). We could also record CPL of (*R,R*)-**1** in dioxane, thus obtaining a g_lum_ value of 4 × 10^−4^ at 550 nm, which is in the range of simple organic molecules. Moreover, ECD and CPL of the opposite enantiomer were also recorded in both pure dioxane and 99.5% water mixture to check the absence of lineal interferences. In dioxane we obtained a mirror CPL spectra to the previous one (Figure S7), thus confirming the value of g_lum_ obtained is attributed to compound (*R,R*)-**1** emission, discarding any possible artifacts (Riehl and Richardson, 1977). However, the experiment in 99.5%water solution afforded a CPL spectra with the same sign to that obtained for the (*R,R*)-**1** enantiomer. With this experiment we can asseverate that the ΔI emission obtained in almost neat water solution was due to an artifact promoted by linear polarization contributions and not actually due to circularly polarized emission (See Figure S8). Moreover, ECD and CPL spectra of compound (*R,R*)-**1** in different solvents were measured (Figure S9) and, in both cases, we obtained maximum values of dissymmetry factors when DMSO was used, with a *g*_abs_ = 2 × 10^−3^ and a *g*_lum_ = 1 × 10^−3^.

Geometry of compound **1** was also optimized by DFT calculations and the corresponding UV-vis and ECD spectra were also simulated with the aid of TD-DFT (See Figures S10–S13 and Tables S1, S2). The calculated ECD signals matched well with experimental ones, as can be seen in Figure S12.

**Figure 6:**
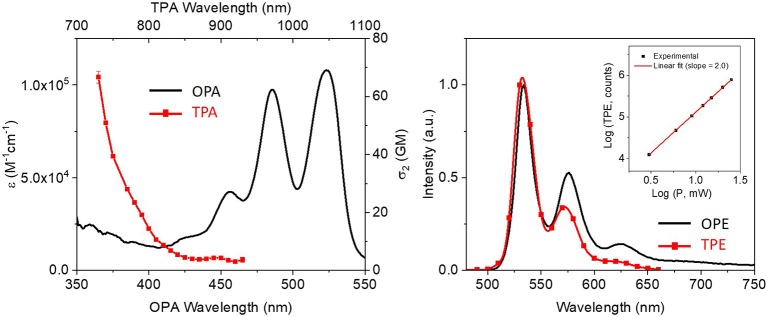
Comparison between one and two-photon absorption and emission of (*R,R*)-**1** in dioxane at *ca*. 10^−6^ M: the left plot shows the one-photon absorption (OPA, black) and two-photon absorption (TPA, red) spectra and the right plot shows the one-photon emission (OPE, black) and two-photon emission (TPE, red). The inset on the right plot represents the Log-Log plot of the photon counts due to non-linear emission as a function of the excitation power. A slope of 2 confirms the biphotonic nature of the process.

In addition to ECD and CPL, two-photon absorption (TPA) and emission (TPE) of a 10^−6^M solution of compound (*R,R*)-**1** in dioxane were analyzed (Figure 6). The emission spectrum under two-photon excitation at 730 nm (red dots in Figure 6B) is similar to the one obtained under one-photon excitation at 456 nm (solid black line). The maximum value of TPA cross-section (σ_2_) was 70 GM at 730 nm, which corresponds to a one-photon energy of 365 nm. The observed blue shifted two-photon absorption with respect to the one-photon absorption maxima is typical of the PDI monomer. This particular spectral shape is attributed to the centrosymmetric structure of the PDI core that causes the TPA under the intense S_0_−*S*_1_ transition to be quasi-forbidden by symmetry. The TPA cross-section is about one order of magnitude higher than that reported for unsubstituted PDIs (Pagoaga et al., 2016). Core substituted PDIs with strong electron donor or electron acceptor groups can have similar TPA values. Remarkably, although the cross-section value is 10 times lower than the one described for our previously reported superhelicene (Figure 1, top middle) (Cruz et al., 2018a) it is only half of the ones described for both nanoribbon (Cruz et al., 2018b) and the triskelion-shape nanographenes (Cruz et al., 2019) (see Figure 1, top left and top right, respectively) but with a simpler and more versatile structure. Due to the order of magnitude lower emission yield and the low concentration of the aggregate in water a reliable estimate of the TPA cross-section of the aggregate was not possible.

## Materials and Methods

### Synthesis of Compound (*R,R*)-1

Compound **1** was prepared according to the protocol previously described by the group of Park et al. (2017). Thus, commercially available (*1R, 2R*)-(–)-1,2-cyclohexanediamine (28.4mg, 0.25 mmol), anhydride **2** (Che et al., 2007) (315 mg, 0.55 mmol) and imidazole (6g) as a solid solvent were heated at 140°C under nitrogen atmosphere for 1 h. Then, 1M HCl was poured into the flask and the formed precipitate was filtrated. The product was purified by flash chromatography in ethyl acetate to afford derivative (*R,R*)-**1** in a 17% yield. Spectroscopic data are detailed in the SI.

### Absorbance and Emission Measurements

A JASCO V-540 spectrophotometer was used to record the linear absorption spectra. The fluorescence spectra were recorded using a Horiba Jobin Yvon Fluorlog 3-22 Spectrofluorimeter with a xenon lamp of 450 W. All the spectra were collected at μM concentration in spectroscopic grade solvents. The fluorescence quantum yields were measured at 492 nm using Rhodamine 6G in methanol as a standard (Φ_F_ = 0.94).

### TPA Measurements

The TPA spectra were measured by the two-photon induced fluorescence (TPF) method using a Ti:sapphire laser (Tsunami BB, Spectra-Physics, 710–990 nm, 1.7 W, 100 fs, 82 MHz). A modified setup that follows the one described by Xu and Webb was used to estimate the TPA cross-section in the 710–920 nm region (Xu and Webb, 1996). Two different standards (Rhodamine 6G in methanol and Fluorescein pH11) where used to account for the collection efficiency and excitation pulse characteristics (de Reguardati et al., 2016). The two-photon absorption cross-section was calculated from the following equation:

$$\begin{array}{l} \sigma_{2}={\left(\frac{F_{2}}{\phi Cn}\right)}_{s}{\left(\frac{\phi Cn\sigma_{2}}{F_{2}}\right)}_{ref} \end{array}$$

where *F*_2_ stands for two-photon induced fluorescence intensity, Φ is the one-photon excited fluorescence quantum yield, *n* refers to the refractive index in solution, *C* is the concentration and *s* and *ref* are relative to the sample and the TPA standard used as reference, respectively. The emission intensity dependence on the excitation power was checked to be quadratic as expected for a biphotonic process. The two-photon emission was measured within a narrow wavelength bandwidth centered at 580 nm selected by the H20Vis Jobin Yvon monochromator placed at the entrance of a PMC-100-4 photomultiplyer tube (Becker and Hickl GmbH). The integrated intensity over the entire emission band was extrapolated using the emission spectra corrected by the detector sensitivity.

### CD and CPL Measurements

ECD and CPL experiments were carried out in an Olis DSM172 spectrophotometer equipped with a Xe lamp of 150W. The spectra were recorded at 2.5 × 10^−5^ M concentrations in HPLC grade solvents and at room temperature. For ECD measurements, a fixed slit-width of 1 mm and 0.1 s of integration time were selected and the ECD spectra shown in Figure 5 and Figures S7–S9 are average spectra calculated from 30 scans. In CPL measurements, a fixed wavelength by a dual excitation of 402 and 420 nm provided by two LED sources was used. CPL spectra in Figure 5 and Figures S7, S8 were collected by averaging 200 scans and with 1s of integration time.

### Theoretical Calculations

Geometry calculation of (*R,R*)-**1** was carried out by DFT methods using the Gaussian 09 software (Frisch et al., 2009). The optimizations were carried out at the CAM-B3LYP/6-31G(d,p) theory level for C, H, N, and O atoms in dichloromethane. The solvent was implemented by using the polarizable continuum model with the integral equation formalism (IEFPCM) available in Gaussian 09. Frequency analysis were performed to confirm that the geometries optimized corresponded to energy minima.

## Data Availability Statement

All datasets generated for this study are included in the article/Supplementary Material.

## Author Contributions

PR and AO carried out the synthesis and ECD and CPL measurements. EM was responsible for the linear and non-linear optical characterization, in addition to writing and editing tasks. IM performed the optical characterization and corresponding data analysis. AC performed DFT calculations. MR AC, JC, EM, and DM were responsible of the management, methodology, resources and writing—review and editing.

## Conflict of Interest

The authors declare that the research was conducted in the absence of any commercial or financial relationships that could be construed as a potential conflict of interest. The handling Editor declared a past co-authorship with several of the authors PR, MR, JC, AC, and DM.
