# Supplementary material for: Simple Perylene Diimide Cyclohexane Derivative With Combined CPL and TPA Properties
Source: Front Chem. 2020 Apr 21;8:306. doi: 10.3389/fchem.2020.00306 (PMC7186504; doi:10.3389/fchem.2020.00306)
Supplement: Supplementary file 1 [file Data_Sheet_1.pdf]

Supplementary Material

Simple perylene diimide cyclohexane derivative with combined CPL and TPA properties

Pablo Reiné,[1] Ana M. Ortuño,[1] Inês F. A Mariz,[2] María Ribagorda,[2] Juan M. Cuerva,[1] Araceli G. Campaña,[1] Emerlinda Maçôas,[2] Delia Miguel[4]

[1] Departamento de Química Orgánica, Facultad de Ciencias, Unidad de Excelencia Química Aplicada a Biomedicina y Medioambiente (UEQ). Universidad de Granada. Avda. Fuentenueva, s/n, E-18071 Granada, Spain.

[2] Centro de Química Estrutural, Instituto Superior Técnico, Universidade de Lisboa, Av. Rovisco Pais, 1, 1049-001 Lisboa, Portugal.

[3] Departamento de Química Orgánica, Facultad de Ciencias, C.U. Cantoblanco, Universidad Autónoma de Madrid, Spain

[4] Departamento de Fisicoquímica, Facultad de Farmacia, UEQ, University of Granada, C.U. Cartuja. Granada, Spain,

*** Correspondence:** Delia Miguel: dmalvarez@ugr.es,
Ermelinda Maçôas: ermelinda.macoas@tecnico.ulisbo.pt

Table of contents

1 **SYNTHETIC PART** 3

1.1 General details 3

1.2 Synthesis and characterization of (R,R)-1 3

Supplementary Scheme 1. Synthesis of *(R,R)*-**1**. 3

1.3 ^{1}H-NMR and ^{13}C-NMR spectra of *(R,R)*-1 4

Supplementary Figure 1. ^{1}H-NMR spectra of compound *(R,R)*-**1** in $CDCl_3$ at 400 MHz. 4

Supplementary Figure 2. ^{13}C-NMR spectra of compound *(R,R)*-**1** in $CDCl_3$ at 101 MHz. 4

2 **LINEAR OPTICAL PROPERTIES** 5

Supplementary Figure 3. Molar absorptivity (ε, $M^{-1}cm^{-1}$) and emission quantum yield (ϕ_f) of *(R,R)*-**1** in different solvents.. 5

Supplementary Figure 4. Normalized UV-vis absorbance (left) and fluorescence spectra (right, λ_{exc} = 485 nm) of *(R,R)*-**1** in different solvents at concentrations of *ca.* 10^{-6}M. 5

Supplementary Figure 5. Solvatochromic effects on the absorption, excitation and emission spectra of compound *(R,R)*-**1** in selected solvents: dioxane, DMSO and water. 6

Supplementary Figure 6. (*R*,*R*)-**1** concentration effect in the formation of the aggregates in water:dioxane mixtures with more than 94% of water followed by (a) the absorption and (b) the emission spectrum upon excitation at λ_{ex}=485 nm........6

3 **CHIROPTICAL PROPERTIES**........7

Supplementary Figure 7. ECD and CPL spectra of 2.5x10^{-5}M solutions of both enantiomers compound **1** in dioxane. 7

Supplementary Figure 8. CPL spectra of 2.5x10^{-5}M solutions of both enantiomers compound **1** in 99.5% water. 7

Supplementary Figure 9. (a) ECD and (b) CPL spectra of 2.5x10^{-5}M solutions of compound (*R*,*R*)-**1** in different solvents 8

4 **COMPUTATIONAL METHODS** 8

4.1 Geometry optimizations........8

Supplementary Figure 10. Side and top view of optimized geometry of *M*-**1** 9

Supplementary Table 1. Atomic coordinates for the DFT calculated structure of *M*-**1**. 12

4.2 UV-Vis and CD spectra calculations 12

Figure S11. Experimental UV-Vis spectrum and calculated UV-Vis spectrum of *M*-**1**. 13

Figure S12. Experimental CD spectrum (top) and calculated CD spectrum (bottom) of *M*-**1**.....13

4.3 Analysis of the first excited states 14

Figure S13. Calculated frontier orbitals contributing to the lowest energy excited stated of *M*-**1** 14

Supplementary Table 2. Orbital contribution to the first excited state of 14

5 **REFERENCES** 15

1 SYNTHETIC PART

1.1 General details

All reagents and solvents (CH_2Cl_2, EtOAc, hexane, THF) were purchased from standard chemical suppliers and used without further purification. Dry THF was freshly distilled over Na/benzophenone. Thin-layer chromatography analysis was performed on aluminium-backed plates coated with silica gel 60 (230-240 mesh) with F254 indicator. The spots were visualized with UV light (254 nm and 360 nm). Chromatography purifications were performed with silica gel 60 (40-63 μm). ^{1}H and ^{13}C NMR spectra were recorded on Varian 400 MHz spectrometers, at a constant temperature of 298 K. Chemical shifts are reported in ppm using residual solvent peak as reference (CDCl3: δ = 7.26 ppm). Data are reported as follows: chemical shift, multiplicity (d: doublet, multiplet), coupling constant (*J* in Hz) and integration; ^{13}C NMR spectra were recorded at 101 MHz using broadband proton decoupling and chemical shifts are reported in ppm using residual solvent peaks as reference ($CDCl_3$: δ = 77.16 ppm). Carbon multiplicities were accomplished by DEPT techniques. High-resolution mass spectra (HRMS) were recorded using ESI mass spectrometry carried out on a Waters Xevo G2-XS QTof mass spectrometer.

1.2 Synthesis and characterization of (R,R)-1

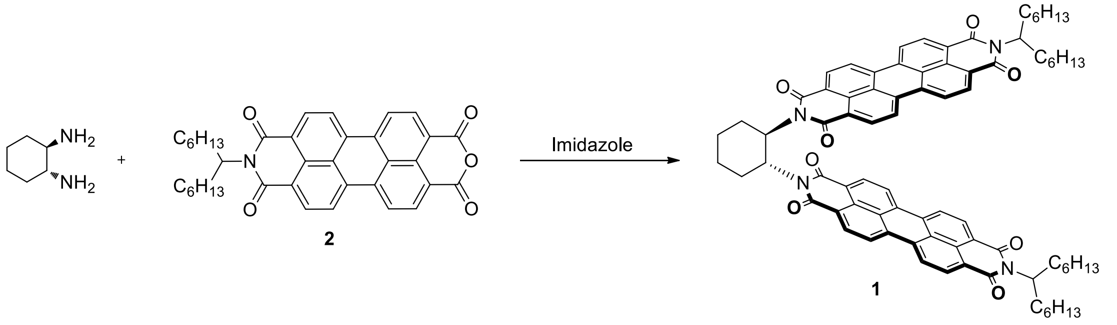

Supplementary Scheme 1. Synthesis of *(R,R)*-**1**.

Compound *(R,R)*-1. (1*R*, 2*R*)-1, 2-cyclohexanediamine (28 mg, 0.242 mmol), **2** (Che et al., 2007) (306 mg, 0.533 mmol), and imidazole (6 g) were added to a two-neck flask. The flask was heated to 140ºC under a nitrogen atmosphere for 1 h. Then, HCl solution was poured into the flask, the precipitate was filtered and purified by column chromatography (ethyl acetate as eluent) to obtain a red solid (80 mg, 27% yield). **^{1}H NMR** (400 MHz, $CDCl_3$) δ 8.57 (d, *J* = 8.1 Hz, 2H), 8.46 – 8.38 (m, 4H), 8.37 – 8.30 (m, 6H), 8.21 (d, *J* = 8.1 Hz, 2H), 8.15 (d, *J* = 8.1 Hz, 2H), 6.41 – 6.35 (m, 2H), 5.09 – 4.99 (m, 2H), 2.83 – 2.73 (m, 2H), 2.20 – 1.96 (m, 8H), 1.86 – 1.71 (m, 6H), 1.38 – 1.07 (m, 32H), 0.82 – 0.71 (m, 12H). **^{13}C NMR** (101 MHz, $CDCl_3$) δ 163.8 (C), 163.7 (C), 134.21 (C), 134.17 (C), 131.5 (CH), 131.3 (CH), 130.9 (CH), 129.4 (C), 129.3 (C), 126.2 (C), 126.1 (C), 124.0 (C), 123.3 (CH), 123.1 (C), 122.9 (CH), 122.82 (CH), 122.77 (CH), 122.6 (CH), 54.8 (CH), 53.4 (CH), 32.4 (CH_2), 31.8 (CH_2), 29.3 (CH_2), 27.0 (CH_2), 25.7 (CH_2), 22.7 (CH_2), 14.1 (CH_3). **HRMS (ES):** *m/z* $[M+Na]^+$ calcd for $C_{80}H_{80}N_4O_8Na$: 1247.5874; found: 1247.5859.

1.3 ^{1}H-NMR and ^{13}C-NMR spectra of *(R,R)*-1

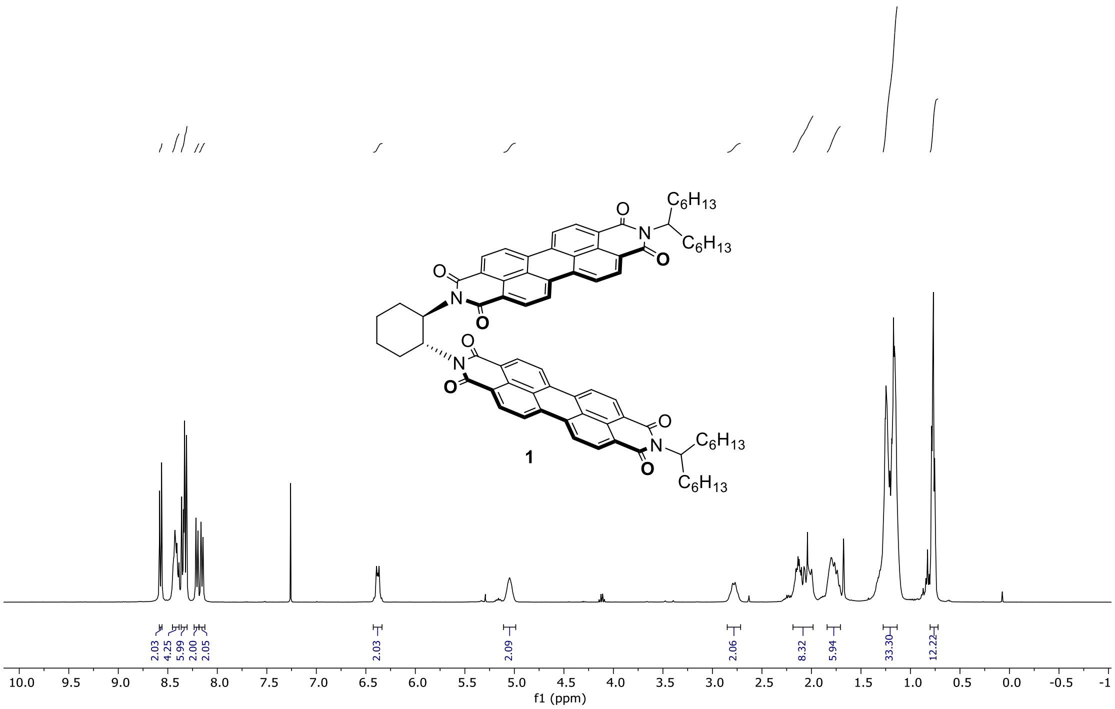

Supplementary Figure 1. ^{1}H-NMR spectra of compound *(R,R)*-**1** in $CDCl_3$ at 400 MHz.

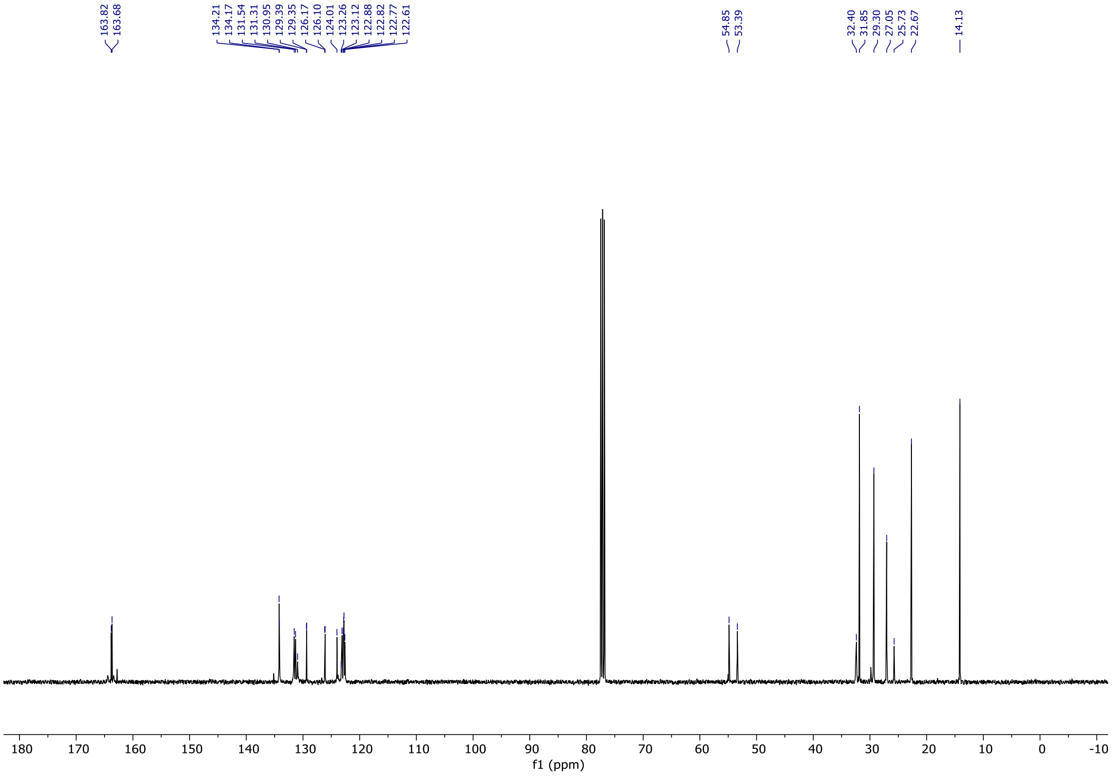

Supplementary Figure 2. ^{13}C-NMR spectra of compound *(R,R)*-**1** in $CDCl_3$ at 101 MHz.

2 LINEAR OPTICAL PROPERTIES

The UV-Vis spectroscopic properties of *(R,R)*-**1** were studied in a range of spectroscopic grade solvents at micromolar concentrations: benzene (Bz), toluene (Tol), dioxane (Diox), chloroform ($CHCl_3$), tetrahydrofuran (THF), dichloromethane (DCM), acetonitrile (ACN), acetone (Ace), Dimethylsulfoxide (DMSO), water a(H_2O)

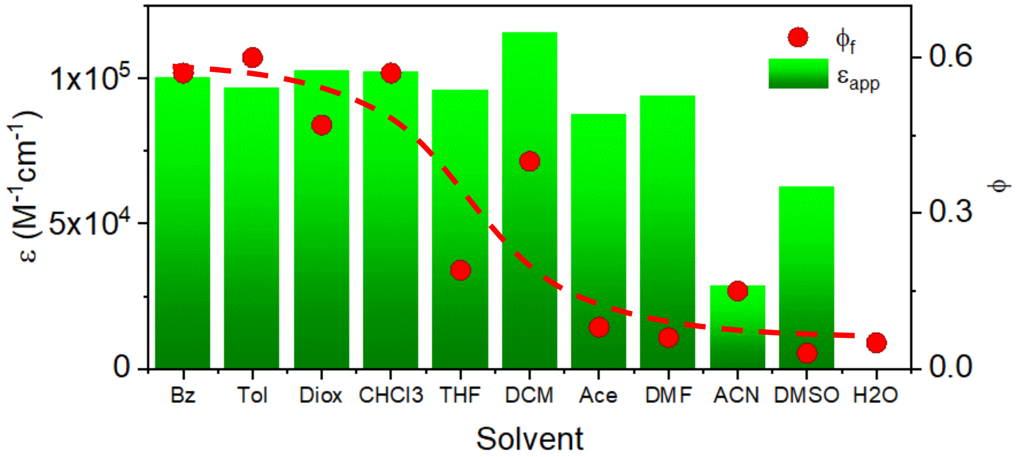

Supplementary Figure 3. Molar absorptivity (ε, $M^{-1}cm^{-1}$) and emission quantum yield (ϕ_f) of *(R,R)*-**1** in different solvents. A dashed line is included for guiding the eye towards the trend in the emission quantum yield. For THF, Ace, ACN, DMF, and DMSO due to the formation aggregates only an apparent molar absorptivity could be estimated based on the overall concentration of the compound.

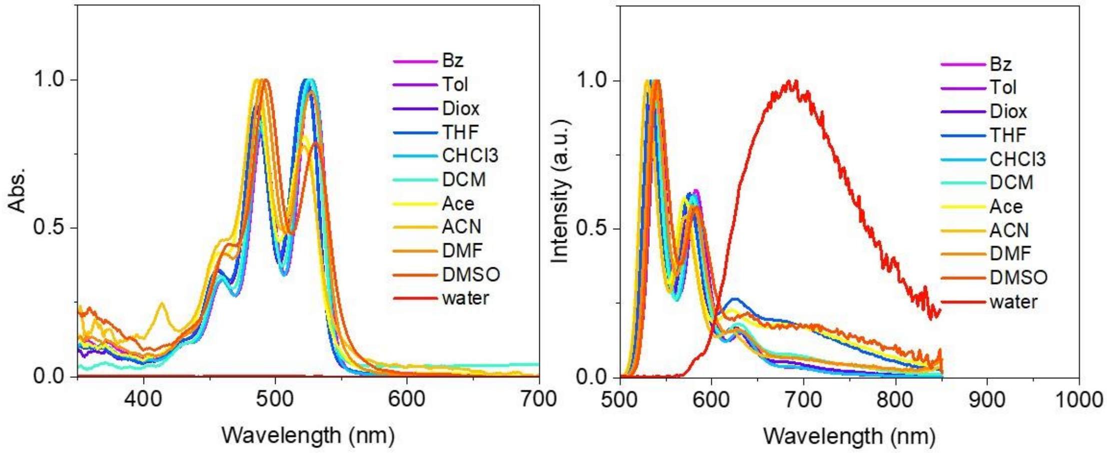

Supplementary Figure 4. Normalized UV-vis absorbance (left) and fluorescence spectra (right, λ_{exc} = 485 nm) of *(R,R)*-**1** in different solvents at concentrations of *ca.* 10^{-6}M.

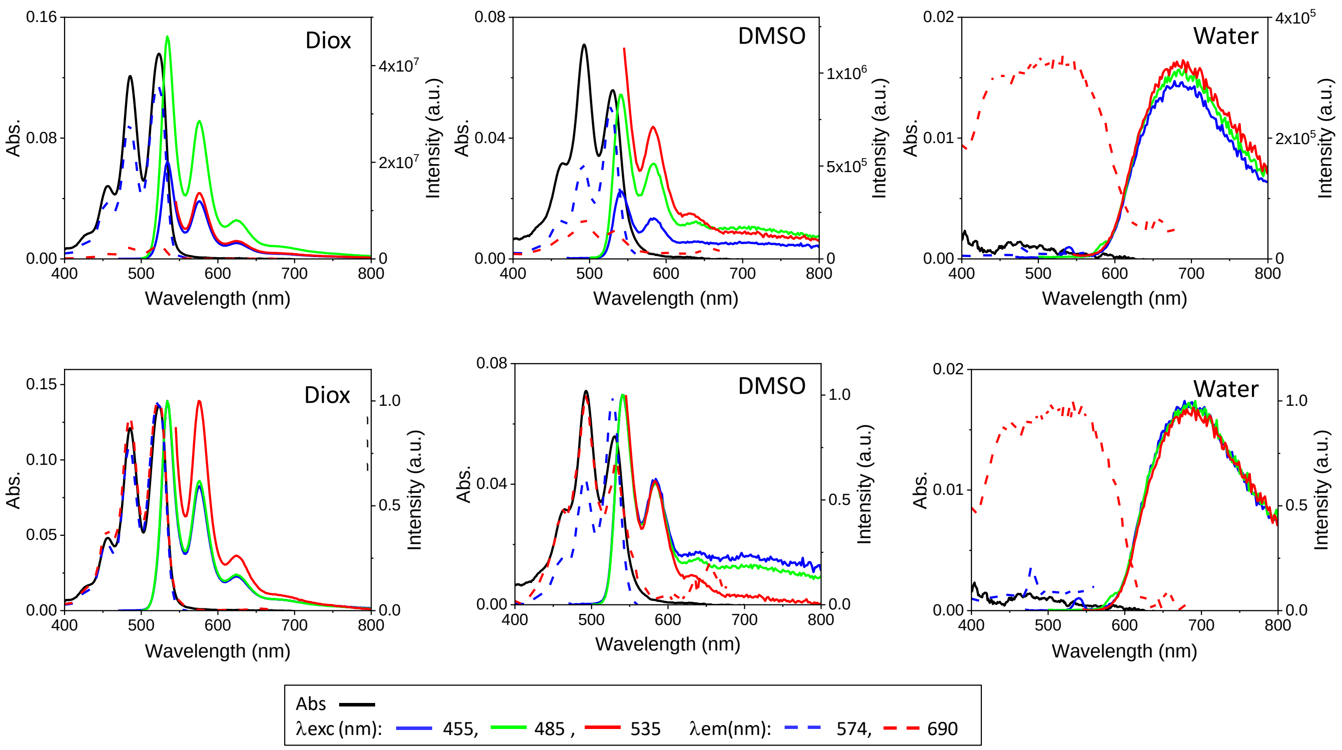

Supplementary Figure 5. Solvatochromic effects on the absorption (full line), excitation (dash line) and emission (full line) spectra of compound *(R,R)*-**1** in selected solvents: (a) dioxane, (b) DMSO and (c) water. Absolute absorption and intensities are shown in the upper plots while the lower plots show normalized values for an easier identification of the overlap between the spectra collected/excited at

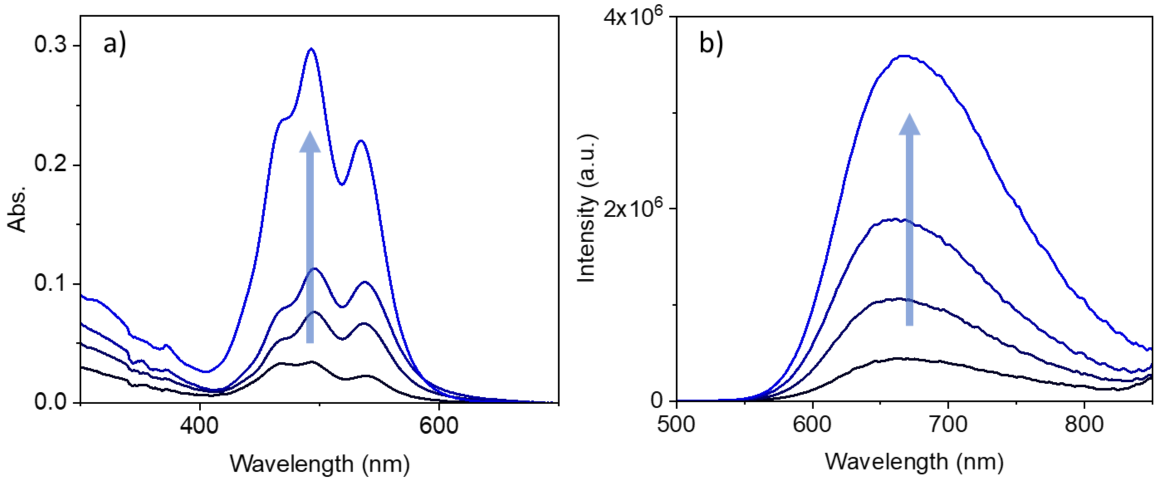

different wavelengths.

Supplementary Figure 6. (*R*,*R*)-**1** concentration effect in the formation of the aggregates in water:dioxane mixtures with more than 94% of water followed by (a) the absorption and (b) the emission spectrum upon excitation at λ_{ex}=485 nm. The concentration ranges from 3.5-13 μM.

3 CHIROPTICAL PROPERTIES

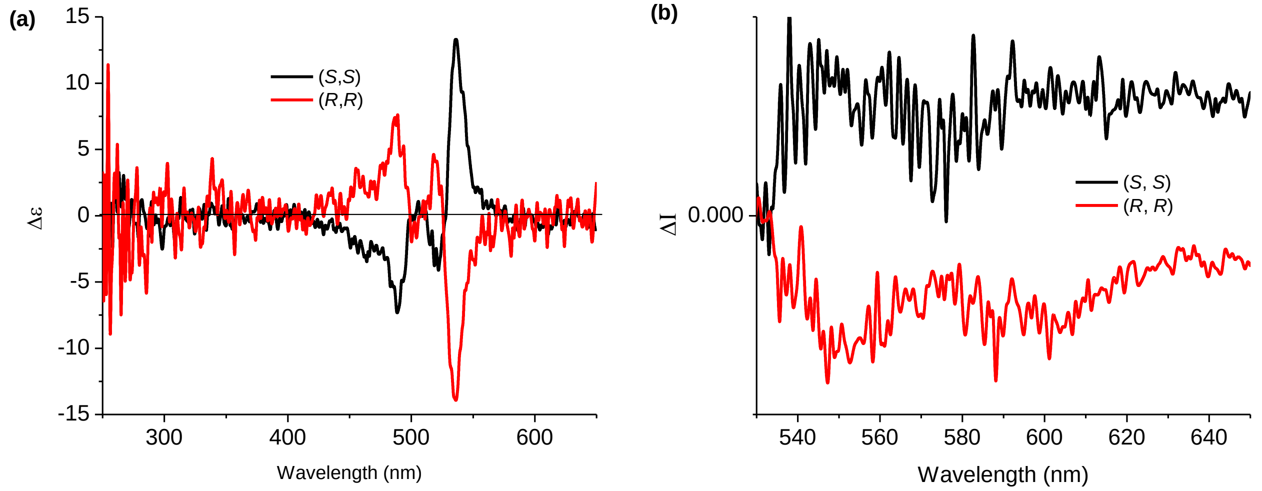

Supplementary Figure 7. ECD and CPL spectra of 2.5x10^{-5}M solutions of both enantiomers compound **1** in dioxane.

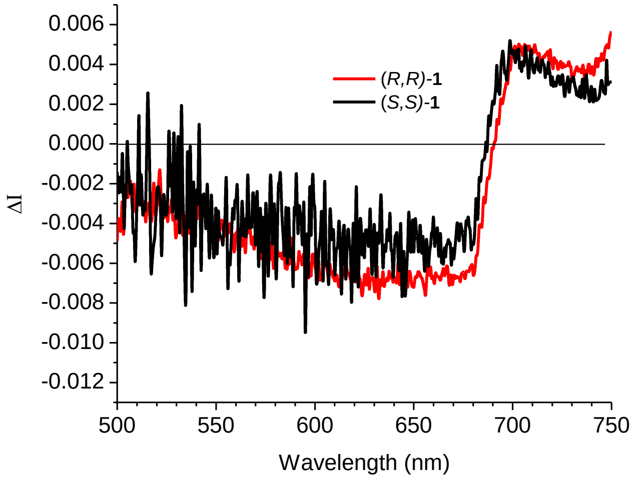

Supplementary Figure 8. CPL spectra of 2.5x10^{-5}M solutions of both enantiomers compound **1** in 99.5% water.

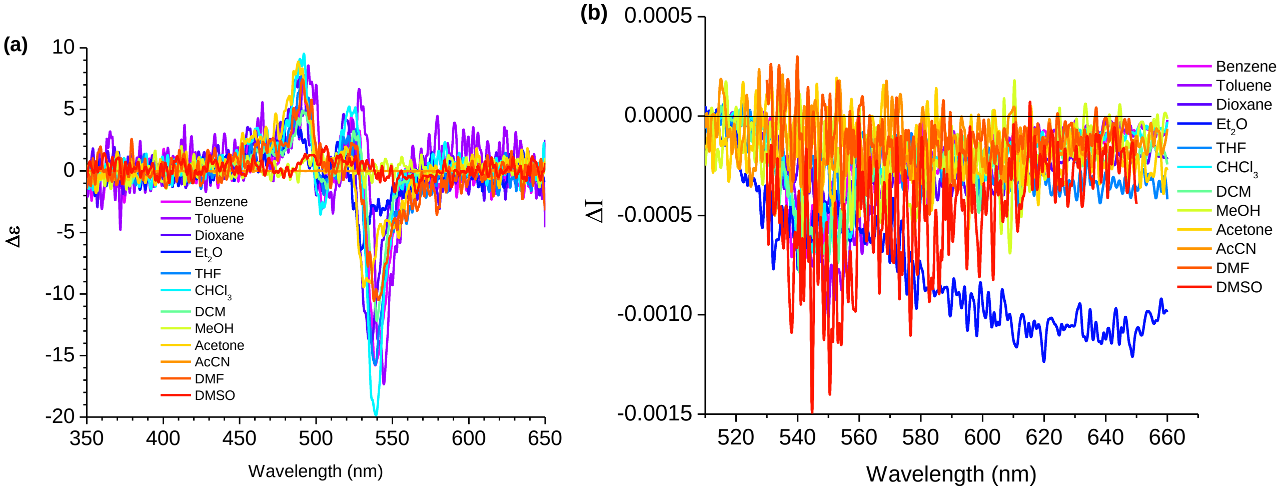

Supplementary Figure 9. (a) ECD and (b) CPL spectra of $2.5x10^{-5}$M solutions of compound (*R*,*R*)-**1** in different solvents

4 COMPUTATIONAL METHODS

4.1 Geometry optimizations

Geometry calculation of the **1** was carried out by DFT methods using the Gaussian 09 software.[S1] The optimizations were carried out at the CAM-B3LYP/6-31G(d,p) theory level for C, H, N and O atoms in dichloromethane. The solvent was implemented by using the polarizable continuum model with the integral equation formalism (IEFPCM) available in Gaussian 09 (Frisch, 2009) Frequency analysis were performed to confirm that the geometries optimized corresponded to energy minima. The atomic coordinates of the calculated structure are shown in Table S1. Colour coding: C, gray; H, white; O, red; N, blue.

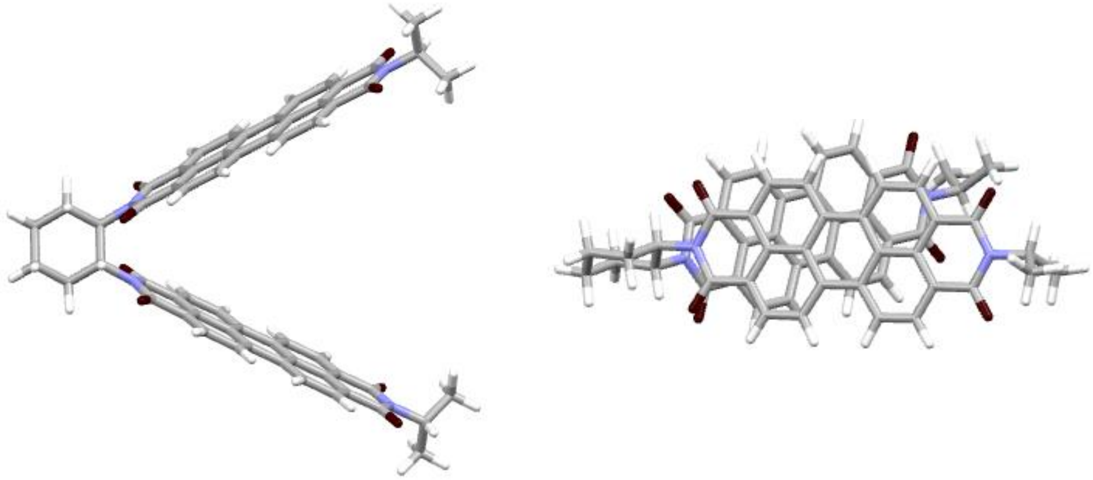

Supplementary Figure 10. Side (left) and top (right) view of optimized geometry of *M*-**1**

Atom	X	Y	Z
C	-0.58751700	8.61513200	0.48854600
C	0.58734500	8.61512700	-0.48865700
C	1.44211000	7.35825200	-0.31952300
C	0.59071800	6.09512500	-0.49461200
C	-0.59093200	6.09514300	0.49453300
C	-1.44230800	7.35827700	0.31938900
C	-3.22154500	0.94068700	2.67517900
C	-2.51820100	2.14857500	2.70972500
C	-2.35697500	2.89235300	1.56452000
C	-2.90939500	2.43634300	0.35046700
C	-3.62198600	1.21113300	0.30335400
C	-3.77578700	0.45435800	1.50243600
C	-2.75149000	3.21354000	-0.81503700
C	-3.29423700	2.78125000	-2.00195200
C	-3.99142600	1.57038200	-2.06144600
C	-4.16438400	0.77394800	-0.94131400
C	-4.88732200	-0.50789800	-0.99395800
C	-5.04411800	-1.26299500	0.20593800
C	-4.51652000	-0.81733600	1.45379900
C	-5.74489500	-2.49496300	0.15639800
C	-5.92160200	-3.26099500	1.32661300
C	-5.41138800	-2.80971600	2.52099200
C	-4.71511400	-1.59859200	2.58032400
C	-5.41905600	-1.00740700	-2.17145800
C	-6.10483700	-2.22562300	-2.20964300
C	-6.27116300	-2.96640900	-1.06353600
C	-1.59917800	4.16105400	1.62552000
N	-1.40368400	4.85840400	0.42411800
C	-2.00885400	4.49356600	-0.77972300
C	-7.00045500	-4.25334100	-1.12935900

N	-7.15217100	-4.97361400	0.05408700
C	-6.65043200	-4.54793300	1.28910400
O	-6.80811900	-5.21510100	2.29962300
O	-7.45416400	-4.67066300	-2.18384800
O	-1.15257900	4.58318500	2.67873900
O	-1.91575300	5.19960900	-1.77114000
C	-7.88214700	-6.27034800	0.02743500
C	3.22185200	0.94093900	-2.67526600
C	2.51853500	2.14884100	-2.70983900
C	2.35703900	2.89248300	-1.56458400
C	2.90927100	2.43638400	-0.35047900
C	3.62188400	1.21118600	-0.30334900
C	3.77576400	0.45443800	-1.50243800
C	2.75111000	3.21345700	0.81507300
C	3.29351200	2.78099400	2.00208100
C	3.99076300	1.57016400	2.06158100
C	4.16419000	0.77397000	0.94135100
C	4.88754300	-0.50764600	0.99392300
C	5.04411800	-1.26288600	-0.20591300
C	4.51620900	-0.81742100	-1.45371300
C	5.74497900	-2.49480800	-0.15637200
C	5.92127100	-3.26109200	-1.32648400
C	5.41051700	-2.81013600	-2.52075400
C	4.71425300	-1.59900900	-2.58010500
C	5.41988300	-1.00680300	2.17129900
C	6.10581900	-2.22493100	2.20946900
C	6.27171700	-2.96597500	1.06346700
C	1.59912800	4.16111900	-1.62558100
N	1.40349200	4.85840400	-0.42416800
C	2.00859200	4.49354800	0.77970200
C	7.00109700	-4.25285800	1.12929500
N	7.15243600	-4.97336200	-0.05405900
C	6.65018900	-4.54798100	-1.28897400

O	6.80749400	-5.21537100	-2.29940600
O	7.45518800	-4.66994400	2.18371200
O	1.15253900	4.58324000	-2.67881000
O	1.91552900	5.19962900	1.77109400
C	7.88244700	-6.27007400	-0.02739200
C	-7.15303900	-7.31027500	-0.81856100
C	-9.34588700	-6.08692200	-0.36307900
C	7.15358300	-7.30989000	0.81895300
C	9.34628100	-6.08655900	0.36273200
H	-0.20504900	8.66334700	1.51587500
H	-1.20767800	9.50454600	0.34110200
H	1.20752500	9.50452600	-0.34120500
H	0.20487200	8.66336400	-1.51598500
H	1.90551300	7.35502500	0.67007400
H	2.24690800	7.33883200	-1.06095900
H	0.18975700	6.09169800	-1.50420200
H	-0.19001300	6.09176500	1.50413800
H	-1.90567500	7.35506100	-0.67022200
H	-2.24713400	7.33887600	1.06079600
H	-3.32047800	0.38817200	3.59988400
H	-2.09049800	2.51086600	3.63676100
H	-3.16991000	3.38999000	-2.88939700
H	-4.39758200	1.26879200	-3.01767000
H	-5.55542900	-3.40672800	3.41334500
H	-4.33025000	-1.28465600	3.54133700
H	-5.31169000	-0.45966400	-3.09813200
H	-6.51277400	-2.59957800	-3.14100100
H	-7.84880700	-6.59672200	1.06481600
H	3.32102900	0.38855100	-3.60002000
H	2.09103000	2.51122900	-3.63692800
H	3.16890200	3.38959000	2.88958500
H	4.39659100	1.26841000	3.01789200
H	5.55415300	-3.40738800	-3.41301100

H	4.32894100	-1.28534500	-3.54102700
H	5.31290900	-0.45883800	3.09788700
H	6.51419400	-2.59862700	3.14074000
H	7.84884900	-6.59661300	-1.06471100
H	-6.11449400	-7.41391000	-0.49357200
H	-7.16727900	-7.04805800	-1.87605200
H	-7.64298400	-8.27942600	-0.69280900
H	-9.82587900	-5.34033500	0.27500900
H	-9.44825000	-5.78016500	-1.40359300
H	-9.87236500	-7.03512100	-0.22564700
H	6.11496400	-7.41362600	0.49422900
H	7.16806000	-7.04749300	1.87639500
H	7.64354000	-8.27904000	0.69324400
H	9.82609000	-5.34002500	-0.27555500
H	9.44889500	-5.77969200	1.40318900
H	9.87275300	-7.03475700	0.22527000

Supplementary Table 1. Atomic coordinates for the DFT calculated structure of *M*-**1**.

Zero-point correction = 0.884443 (Hartree/Particle)

Thermal correction to Energy = 0.938636

Thermal correction to Enthalpy = 0.939580

Thermal correction to Gibbs Free Energy = 0.793197

Sum of electronic and zero-point Energies = -3129.216102

Sum of electronic and thermal Energies = -3129.161909

Sum of electronic and thermal Enthalpies = -3129.160965

Sum of electronic and thermal Free Energies = -3129.307348

4.2 UV-Vis and CD spectra calculations

Electronic transitions of compounds **1** was calculated by means of TD-DFT methods using the Gaussian 09 software (Frisch, 2009). For the calculations the CAM-B3LYP/6-31G(d,p) theory level was used and the first 50 excited states were considered. The optimized coordinates shown in Table S1 was used. Calculations were carried out in dichloromethane by using the same polarizable continuum model with the integral equation formalism (IEFPCM) applied for the geometry optimizations.

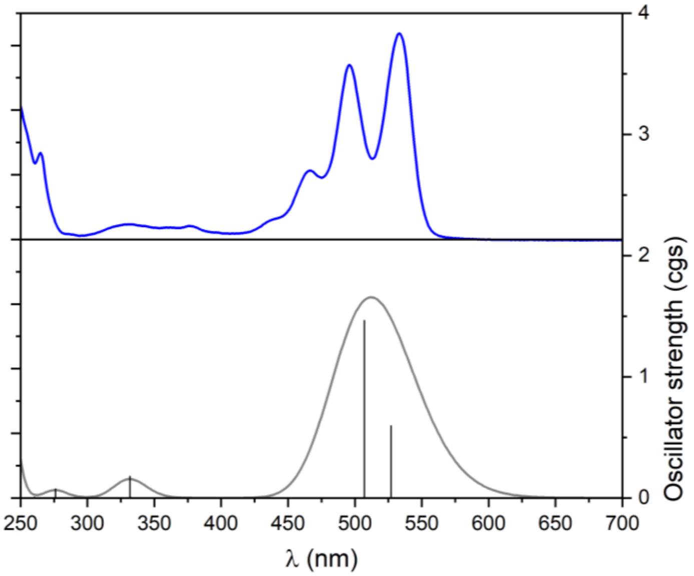

Figure S11. Experimental UV-Vis spectrum (top) and calculated UV-Vis spectrum (bottom) of *M*-**1**. The calculated values have been corrected by −0.3 eV.

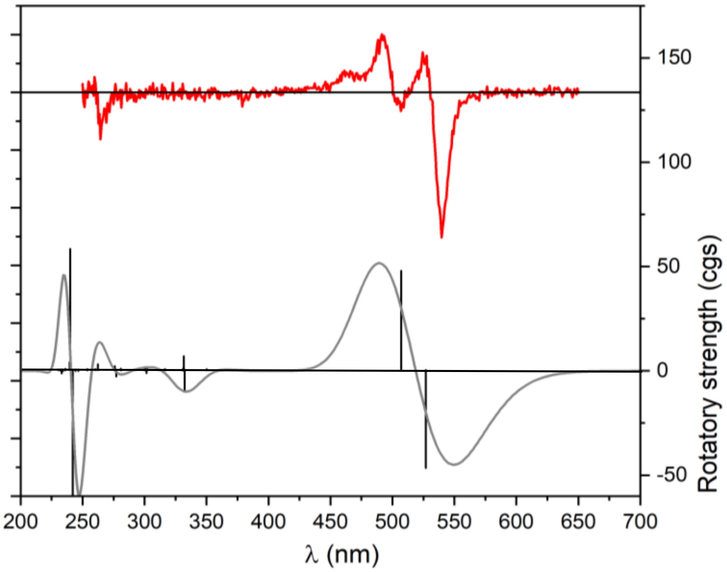

Figure S12. Experimental CD spectrum (top) and calculated CD spectrum (bottom) of *M*-**1**. The calculated values have been corrected by −0.3 eV.

4.3 Analysis of the first excited states

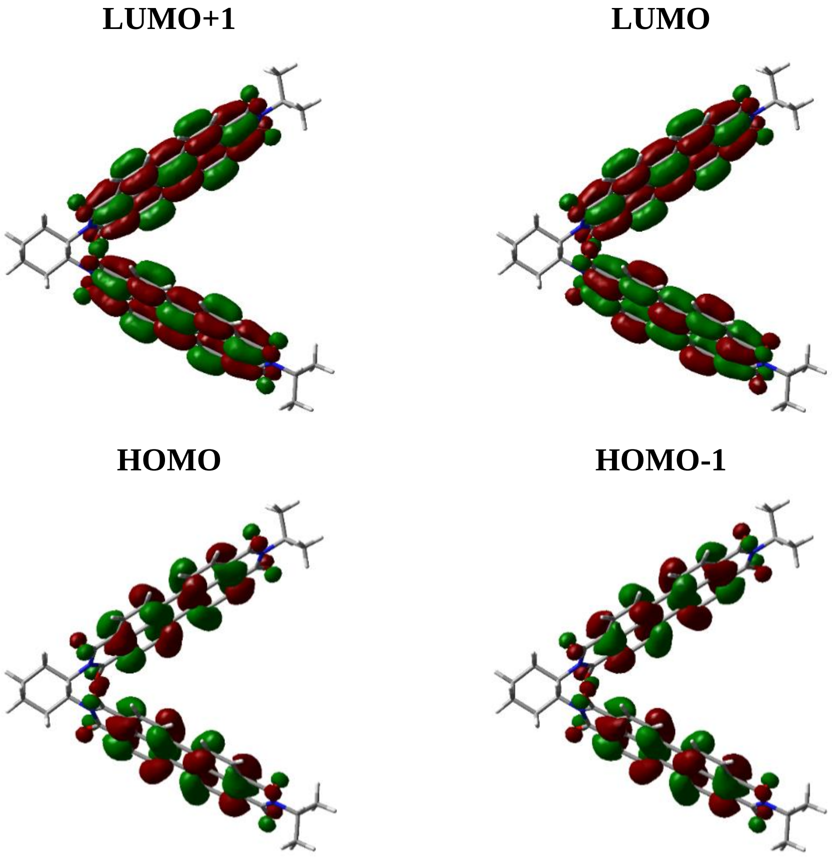

Figure S13. Calculated frontier orbitals contributing to the lowest energy excited stated of *M*-**1** (Surface representation parameters: isovalue, 0.02000; density: 0.00040).

**Orbitals Contributing**	**Contribution to excitation** (Gaussian coefficient)
H → L	53.3% (0.51189)
H−1 → L+1	46.7% (−0.47901)

Supplementary Table 2. Orbital contribution to the first excited state of *M*-**1**.

5 REFERENCES

Che, Y., Datar, A., Balakrishnan, K., and Zang, L. (2007). Single-Molecule Spectroscopy of Interfacial Electron Transfer. Ultralong Nanobelts Self-Assembled from an Asymmetric Perylene Tetracarboxylic Diimide *J. Am. Chem. Soc.* 129, 7234-7235.

Frisch, M. J., Trucks, G.W., Schlegel, H.B., Scuseria, G. E., Robb, M. A., Cheeseman, J. R., et al Gaussian 09 Revision D.01, Gaussian Inc. Wallingford CT, 2009.

.
